# Beyond Rodents: Alternative Animal Models in Colorectal Cancer Research

**DOI:** 10.3390/ijms262210874

**Published:** 2025-11-09

**Authors:** Wei Xiong, Solène Favier, Ting Wu, Frédérique Ponce, Charles Dumontet, Marie Alexandra Albaret, Frédéric Hollande, Jean-Jacques Diaz, Hichem C. Mertani

**Affiliations:** 1 LabEx Dev2CAN, Institut Convergence Plascan, Centre de Recherche en Cancérologie de Lyon, Inserm U1052, CNRS UMR5286, Centre Léon Bérard, Université Claude Bernard Lyon 1, Université de Lyon, CEDEX 08, 69008 Lyon, France; blariotxiong@alumni.sjtu.edu.cn (W.X.); solene.favier@gmail.com (S.F.); wuting1989@swmu.edu.cn (T.W.); charles.dumontet@chu-lyon.fr (C.D.); marie.albaret@lyon.unicancer.fr (M.A.A.); jeanjacques.diaz@lyon.unicancer.fr (J.-J.D.); 2 Plasticity, Heterogeneity and Tumour Microenvironment International Research (PHANTOM) Laboratory, Centre National de la Recherche Scientifique (CNRS) and The University of Melbourne, Melbourne, VIC 3000, Australia; frederic.hollande@unimelb.edu.au; 3 Service de Cancérologie, UR ICE, VetAgro Sup, Université de Lyon, 69280 Marcy-l’Étoile, France; frederique.ponce@vetagro-sup.fr; 4 Hospices Civils de Lyon, 69008 Lyon, France; 5 Department of Clinical Pathology, The University of Melbourne, Melbourne, VIC 3000, Australia; 6 Collaborative Centre for Genomic Cancer Medicine, The University of Melbourne, Melbourne, VIC 3000, Australia

**Keywords:** colorectal cancer, nonrodent animals, model organisms, chorioallantoic membrane model, mammals, translational research, drug resistance

## Abstract

Colorectal cancer (CRC) is the third most common cancer worldwide, imposing a significant burden on public health. Despite the use of various therapeutic strategies, the prognosis for patients with metastatic and drug-resistant CRC remains poor, which underscores the need for further investigations into cancer mechanisms to develop more effective treatments. Rodents, particularly mice, are the most frequently used animal models for CRC research. However, as the demand for more precise simulations and higher ethical standards in animal experimentation grows, the applicability of rodent models may face increasing limitations. This review highlights a variety of non-rodent animals, including model organisms such as zebrafish (*Danio rerio*), fruit flies (*Drosophila melanogaster*), and *Caenorhabditis elegans* (*C. elegans*), as well as the chorioallantoic membrane (CAM) model and mammals such as rabbits (*Oryctolagus cuniculus*), dogs (*Canis lupus familiaris*), and pigs (*Sus scrofa domesticus*), which have been utilized in CRC research. Each of these alternatives offers specific advantages in certain areas of cancer research. Their use has enabled new insights into the mechanisms of carcinogenesis, metastasis, and drug resistance in CRC, as well as the development of novel therapies.

## 1. Introduction

Colorectal cancer (CRC), a type of malignant tumor that occurs in the colon and rectum, imposes a significant burden on human health. According to Global Cancer Statistics 2022 (GLOBOCAN 2022) [1], CRC ranks as the third most common cancer globally, with 1,926,118 new cases yearly, accounting for 9.6% of all newly diagnosed cancers. It is also the second leading cause of cancer-related deaths, with 903,859 annual deaths, accounting for 9.3% of all cancer deaths [1]. There is an overall increasing trend in CRC incidence, with an estimated 2.2 million new cases expected by 2030 [2]. The primary source of this increase will be developing countries undergoing rapid economic advancements [3] and the increasing incidence of early-onset CRC in patients under 50 years of age [4,5].

CRC has a complex etiology and numerous risk factors. In 1990, Fearon and Vogelstein proposed a genetic model for colorectal tumorigenesis, suggesting that CRC arises from a sequential accumulation of genetic mutations [6]. After decades of research, several oncogenes and tumor suppressor genes have been identified, and CRC has been classified into four molecular subtypes [7]. In recent years, CRC screening methods, including fecal occult blood tests (FOBTs), colonoscopy, and genetic profiling, have become increasingly widespread, enabling early cancer detection for many individuals. Researchers have also developed comprehensive treatment approaches, including chemotherapy and radiotherapy, which are sometimes combined with targeted therapy or immunotherapy.

However, the five-year survival rate of patients with metastatic cancer is only 15% [8]. Additionally, cancer drug resistance continues to hinder treatment efforts. In light of these challenges, there has been a sustained effort to further elucidate the molecular mechanisms of CRC and to develop more effective therapies [9]. In CRC research, whether in basic studies or translational applications, the selection of appropriate animal models is pivotal, as it directly affects the clinical relevance and reliability of findings. Animal cancer models can be broadly categorized into spontaneous models, induced models, genetically engineered models (GEMs), and transplant models. In terms of species, rodent models, particularly mouse models, are especially prominent [10,11]. While they provide valuable insights into human physiology and disease mechanisms, two key limitations have prompted the exploration of complementary models. First, their evolutionary distance from humans results in notable differences in organ structure and pathological responses compared with those of more clinically relevant large mammals such as dogs and pigs. Second, murine models present logistical challenges, including high maintenance costs, ethical constraints, and difficulties in scaling for high-throughput studies. To address these gaps, researchers are developing a multi-model approach: species with increased clinical translatability (e.g., dogs, rabbits, and pigs) increase pathological relevance, whereas other model organisms, such as zebrafish, fruit flies, and chick embryo chorioallantoic (CAM) membranes, offer cost-effective solutions for genetic screening and large-scale therapeutic testing. This integrated strategy leverages each model’s unique strengths while mitigating individual limitations.

**Figure 1:**
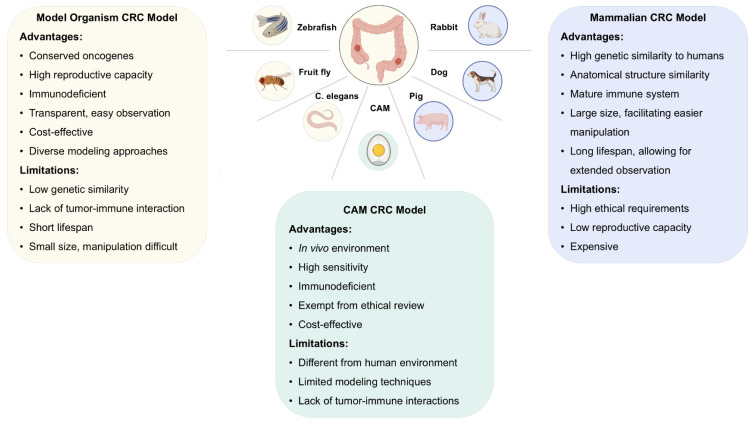
Nonrodent models used in colorectal cancer (CRC): benefits and drawbacks. Nonrodent models for CRC can be classified into three main types: organism-based models, chorioallantoic membrane models, and mammalian models. These models provide valuable insights into human CRC, with each type offering unique advantages and limitations. The choice of model should be based on the specific needs of the experiment. Created in BioRender (https://www.biorender.com/).

This review focuses on various types of non-rodent animal models for CRC, including organism-based models, the CAM model, and less commonly used mammalian models, and discusses their respective advantages and limitations (Figure 1).

## 2. Application of Organism-Based Models in Colorectal Cancer

In the nascent stages of developmental biology research, researchers employed a series of model organisms with relatively simple and conserved genetic information and a limited number of cells as research models to observe developmental patterns and elucidate basic molecular mechanisms. As early as the 17th century, mice were used in experimental studies [12]. In the late 19th century, Oskar Hertwig employed sea urchins to study fertilization and embryonic development [13]. As studies on model organisms can address fundamental questions in numerous areas of the life sciences, their applications extend to a range of biological fields, including oncology. In this section, we focus on three non-rodent model organisms, zebrafish (*Danio rerio*), fruit flies (*Drosophila melanogaster*), and *C. elegans* (*Caenorhabditis elegans*), in CRC research.

### 2.1. Zebrafish (Danio rerio)

The zebrafish is a common tropical fish characterized by its slender body and an adult length of 3–4 cm, and was first utilized in developmental biology. Since the 1980s, researchers have explored the use of zebrafish as a cancer research model. Zebrafish offer several distinct advantages in cancer studies. First, more than 80% of human disease-related genes are present in the zebrafish genome, particularly highly conserved cancer-related genes [14]. Zebrafish also have high reproductive capacity, producing hundreds of eggs at a time, which develop into larvae within 3 days [15], facilitating large-scale experimental studies. Additionally, zebrafish larvae are transparent, making it easy to observe tumor morphology, localization, and progression [16]. Zebrafish are quite cheap to maintain compared with mice, and the experiments are much simpler to perform [17].

There is a certain degree of conservation between zebrafish and human intestines [18]. Both originate from the endoderm and are regulated by similar cytokines, such as bone morphogenetic protein (BMP) and fibroblast growth factor (FGF) [19,20]. Anatomically, the zebrafish digestive tract includes a large-diameter intestinal bulb (similar to the stomach) and a smaller-diameter posterior digestive tract (similar to the intestine). The zebrafish digestive tract also exhibits changes similar to those in mammals, with the intestinal epithelium transitioning from a high epithelium containing secretory cells and goblet cells near the stomach to a low epithelium lacking these cells near the anus [19]. Wang et al. combined morphological and transcriptomic analyses to further validate the similarities between zebrafish and human intestines [21], a finding supported by functional genomics studies indicating a high level of functional conservation between the intestinal epithelial cell transcriptional programs of zebrafish and humans [22]. These studies provide important support for the construction of zebrafish models of CRC.

The methods used to construct zebrafish models of CRC are diverse, with xenotransplantation being one of the more commonly used approaches. Given their lack of an adaptive immune system, small size, and transparency, which facilitate observation, zebrafish are considered one of the most suitable animal models for in vivo xenotransplantation [23]. Zebrafish xenotransplantation, first developed by Lee et al. in 2005 [24], involves the implantation of human tumor fragments into the perivitelline space of zebrafish embryos, allowing the tumors to grow. In the field of CRC, the transplantation of both tumor cell lines and patient-derived tumor cells has now been achieved. These models are applicable not only in preclinical drug trials but also for monitoring drug resistance and formulating personalized treatment strategies for individual patients [25].

Wild-type zebrafish can also develop spontaneous intestinal tumors, which exhibit pathological features similar to those of humans, such as inflammatory infiltration, hyperplasia, and abnormal cellular morphology [26]. However, spontaneous models are not commonly used because of their inefficiency.

The construction of induced models involves the injection of chemical inducers, including 2,4,6-trinitrobenzene sulfonic acid (TNBS) [27], dextran sodium sulfate (DSS) [28], and 7,12-dimethylbenzanthracene (DMBA) [29]. They induce CRC through inflammatory responses; thus, they are crucial for studying the relationship between inflammation and tumorigenesis and can be used to explore protective and promoting factors involved in the inflammation-cancer development process, such as the intestinal epithelial cell regulatory molecule retinoic acid [30] and the gut microbiota [31].

One of the most well-known genetically engineered zebrafish CRC models is the adenomatous polyposis coli (*APC*) mutation model. In humans, *APC* is a crucial tumor suppressor gene located on chromosome 5q21, primarily through its role as a negative regulator of the wingless-type/catenin-beta (WNT/β-catenin) pathway [32]. Individuals with congenital *APC* defects develop familial adenomatous polyposis (FAP), which progresses to CRC [33]. Acquired mutations in this gene are considered important early markers of CRC development [6]. The *APC* gene is widely conserved across species and plays a similar role, making it a key target for constructing CRC models. In zebrafish, *APC* mutations are selected through induced mutagenesis, leading to abnormal gene expression due to premature stop codons [34], and 10–15% of heterozygous mutant zebrafish develop intestinal tumors [34]. Other common gene-editing models include the shock-inducible Cre/Lox-mediated human *kRASG12D* transgenic fish model, in which the abnormal proliferation of the intestinal epithelium leads to cancer [35], and the N-ethyl-N-nitrosourea-induced tumor protein p53 (*TP53*) mutation model [36]. While the genomic profiles of tumors in these models are considerably less complex than those of human CRC, they remain essential tools for dissecting core molecular pathways and providing preliminary insights into targeted therapy responses. However, their limited heterogeneity underscores the need for complementary validation in more representative human-like models.

Since both the transparency and immune deficiency of zebrafish are lost as they mature, Casper zebrafish mutants, which lack melanocytes, have been developed. These fish remain transparent throughout their lives, facilitating the monitoring of tumor growth [37]. Researchers have also induced recombination-activating gene 2 (*RAG2E450fs*) mutant zebrafish, in which the interaction between *RAG2* and trimethylated histone H3 is disrupted. This alteration affects chromatin accessibility and partially impairs V(D)J recombination, resulting in lifelong adaptive immune deficiency in these zebrafish, which facilitates the transplantation of exogenous tumors [38].

### 2.2. Fruit Fly (Drosophila melanogaster)

The fruit fly is an invertebrate model organism first reported by Meigen in 1830 [39], and in the early 20th century, Morgan et al. discovered the laws of genetic linkage and recombination in fruit flies [40]. Fruit flies are small in size, with a body length of 2–2.5 mm, are easy to maintain, reproduce rapidly, have a short life cycle, and have a simple genetic makeup with only four pairs of chromosomes (including one pair of sex chromosomes). These characteristics are key advantages of this species as an important model organism [41]. In addition to genetics, the fruit fly is a crucial cancer research model. Mary Stark first used it to study the etiology of cancer as early as the 1920s [42]. Over nearly a century of research, fruit flies have been shown to share 75% of human disease-related genes and can develop cancer [43]. Fruit fly cancer models are valuable tools for investigating the molecular mechanisms underlying various cancers and for advancing drug development research [44].

Since the late 20th century, when fruit fly intestine somatic cells were first isolated, researchers have recognized them as valuable models for studying CRC [45]. The fruit fly intestine can be divided into the foregut and hindgut, which originate from the ectoderm, and the midgut, which originates from the endoderm. The entire midgut contains a type of intestinal stem cell (ISC) [46,47]. ISCs exhibit remarkable heterogeneity, regenerative capacity, and plasticity and are influenced by multiple signaling pathways, such as the Notch, Janus kinase/Signal transducer and activator of transcription (Jak/Stat), epidermal growth factor receptor (EGFR), Mechanistic Target of Rapamycin (mTOR), and Decapentaplegic/Bone morphogenetic rotein (Dpp/BMP) pathways, which also play roles in maintaining and renewing the human intestine and are involved in CRC development [48,49,50,51]. As a result, current research on fruit fly CRC models has focused primarily on the midgut.

When Mary Stark first used fruit flies to study cancer, she transplanted cancerous tissue from diseased flies into healthy ones and demonstrated the feasibility of transplantation [41]. However, no studies have yet reported successful CRC transplantation in fruit flies. This may be due to the small size and high mobility of fruit flies, combined with their innate immune system’s robust immune rejection of foreign substances [52]. Given the extremely short lifespan of fruit flies, no inducible CRC model has been identified. However, considering their foraging behavior, they may represent a suitable model for studying CRC dietary risk factors.

Gene engineering techniques are the most commonly used methods for constructing fruit fly CRC models, primarily because of the presence of ISCs in the midgut [53]. As mentioned above, ISC division and differentiation are regulated by signaling pathways, and disrupting these pathways through gene editing can lead to excessive ISC proliferation or loss of differentiation, thereby creating CRC models. The most classic model remains the *APC* mutation model, where ISC clones with mutations in both fruit fly *Apc1* and *Apc2* genes (*Apc1Q8* and *Apc2g10* mutants) can develop into multilayered epithelial cells that fuse, resulting in intestinal occlusion and narrowing [54]. These Apc mutant cells also kill surrounding normal cells to facilitate cancer growth [55]. The Ras gene is another common target, where the expression of RasV12 in ISCs, driven by the upstream activation sequence-Gal4 (UAS-Gal4) system, promotes intestinal tumorigenesis in fruit flies [54]. Studies have shown that Notch-deficient ISCs drive tumorigenesis through a triphasic mechanism—stress-primed proliferation, physical competition, and niche signaling hijacking—without requiring additional mutations [56]. While neither Notch loss nor KRAS activation alone is sufficient to drive CRC in humans, these findings highlight how fruit fly models are particularly useful for dissecting early oncogenic events in isolation and for revealing conserved mechanisms of stem cell dysregulation, competitive behavior, and microenvironmental interactions that may precede full malignant transformation. Thus, fruit fly models offer a powerful platform for identifying and mechanistically characterizing fundamental pathways relevant to the initiation of CRC, even if they do not fully recapitulate the multigenic complexity of human tumors.

Recent research has suggested that ISCs also exist in the fruit fly hindgut proliferation zone (HPZ) [57]. Bangi et al. utilized HPZ ISCs to develop a quadruple gene-editing model that simultaneously targeted and edited the *Ras*, *Tp53*, *Pten*, and *Apc* genes. The edited cells exhibited abnormal proliferation and epithelial–mesenchymal transition (EMT) within the fruit fly intestine. Since multigene edited models more closely resemble the actual mutational landscape in humans, this model holds significant potential for studying cancer mechanisms and metastasis, identifying new drug targets, and conducting preclinical experiments [58].

### 2.3. Caenorhabditis elegans (C. elegans)

*C. elegans* is approximately 1 mm in length and resides in humus-rich environments. It was first identified in 1899 by the French scientist Maupas [59] and was established as a prominent model organism in the 1960s [60]. It possesses a simple genome of only 97 Mb but shares 60–80% of disease-related genes with humans, making it highly valuable for manipulation via various genetic tools, such as CRISPR-Cas9 and RNA interference [61,62]. Additionally, the genome of *C. elegans* contains fewer members in the same cancer-related gene families; for example, the mammalian *TP53* gene family has three members (p53, p63, and p73), whereas *C. elegans* possesses a single homolog of this entire family, *Caenorhabditis elegans* p53-like gene-1 (*cep-1*) [63]. This genetic simplicity, with minimal redundancy, makes it easier to observe clear biological effects following gene inactivation, as compensatory mechanisms by paralogous genes are largely absent. Because of its short lifecycle (approximately 2–3 weeks), high reproductive capacity (hermaphrodites produce approximately 250 offspring within 3–4 days) [64], transparency, adaptive immune inefficiency [65], ease of maintenance, and low cost, *C. elegans* can serve as a model organism for addressing fundamental and translational research, such as studying signaling pathways and assessing the efficacy of anticancer drugs [66], in several cancer types. Ten of the 14 cancer hallmarks can be studied in *C. elegans*, including cell proliferation, cell cycle regulation, cellular immortality, and migratory capacity [67]. Research on *C. elegans* has enabled the elucidation of regulatory mechanisms within the cyclin-dependent kinase inhibitor (*CKI*) family [68], of the cyclin-dependent kinase (CDK) phosphorylation controlled by the Wee1 family [69], and of the cell cycle consequences following the loss of the tumor suppressor protein pRb [70], thus highlighting the role of these pathways in cancer development. The migration of distal tip cells during gonad development, involving the matrix metalloproteinases (MMPs) GON-1 and MIG-17, along with UNC-6/netrin and its receptors, has been used as a model to study cancer metastasis because these proteins play similar roles in tumor cell migration [71,72,73]. In therapeutic research, *C. elegans* is utilized to evaluate the effectiveness and toxicity of chemotherapeutic agents such as bleomycin and mitomycin [74,75], to determine the efficacy of targeted therapies, and to identify potential new drug targets [76,77].

Although *C. elegans* cannot model CRC tumorigenesis due to its non-renewing intestine composed of merely 20 cells and lacking stem cells [78,79], the core value of *C. elegans* lies in serving as a valuable complementary model for CRC-associated processes such as cellular aging, host-microbiota interactions, and drug metabolism, leveraging its structural simplicity to advantage. First, owing to its inability to replace damaged cells, *C. elegans* provides a unique opportunity to observe the long-term effects of aging, which is closely linked to cancer development through processes such as DNA damage and altered gene regulation [79]. For example, Wang et al. identified 23 genes that promote intestinal aging in *C. elegans*; some of these genes are abnormally expressed in human CRC and are associated with poor prognosis, highlighting the need for further investigation into their related pathways [80]. Moreover, *C. elegans* feeds on bacteria and can establish its gut microbiota directly from its environment [81]. Researchers can create *C. elegans* with certain microbiota compositions to study their effects on the intestine [82]. Additionally, *C. elegans* can be utilized to explore the mechanisms of drug resistance, such as 5-fluorouracil (5-FU), a common CRC chemotherapy drug. In *C. elegans*, alterations in uridine phosphorylase 1 (*Upp1*) prevent the conversion of 5-FU into its toxic metabolites, resulting in resistance to 5-FU, which is similar to that in humans. Therefore, this model can be utilized to explore potential strategies to overcome drug resistance [83].

## 3. Application of the Chick Embryo Chorioallantoic Membrane (CAM) Model

The chick embryo chorioallantoic membrane (CAM) is rich in capillaries and facilitates gas exchange between the embryo and the external environment [84]. Owing to its abundant vascular network connected to the embryo, the CAM has long been utilized in oncological research: in 1911, Rous et al. transplanted tumors onto the CAM, establishing the first tumor chorioallantoic membrane (TUM-CAM) model [85]. This model combines the advantages of both in vivo and in vitro systems. It can closely mimic an in vivo cancer environment while at the same time being readily available, cost-effective, and suitable for large-scale experiments. The chick embryo is in an immunodeficient state before day 11, allowing for 80% survival of the transplanted cells [86,87]. To date, all established TUM-CAM models are transplant models, including those for neuroblastoma [88], breast cancer [89], and CRC [90]. These models are widely applied in studies of tumor angiogenesis, tumor invasion and metastasis, and tumor drug screening [91]. From an experimental ethics perspective, the chick embryo model holds a unique advantage. The U.S. National Institutes of Health has determined that embryos prior to day 14 of incubation do not experience pain, giving them a favorable status in ethical reviews [92]. In practical applications, however, researchers should adhere to the Replacement, Reduction, Refinement (3R) principles. Furthermore, when human cell transplantation is involved, strict regulatory boundaries must be observed, which explicitly prohibit any contribution of human cells to the germline or their extensive integration into the developing central nervous system of the embryo [93]. The TUM-CAM model of CRC is well developed, with the most common method involving the injection of mouse- or human-derived CRC cell suspensions onto the CAM and the observation of cancer cell growth and neovascularization by using stereoscopic imaging microscopes, computed tomography, and other techniques, sometimes in conjunction with fluorescent proteins to enhance tumor visibility [94,95]. In this model, cold atmospheric plasma (CAP)—a partially ionized gas composed of reactive oxygen and nitrogen species (ROS/RNS), UV photons, and charged particles—has been explored as a novel therapeutic approach. Using CT26 CRC cells, the TUM-CAM model revealed that different CAP devices mediate tumor suppression through distinct mechanisms: the kINPen device induced immunogenic cell death (ICD), characterized by the exposure and release of damage-associated molecular patterns (DAMPs), like calreticulin, ATP, and nuclear High Mobility Group Box 1 (HMGB1), that are normally hidden inside cells but are released during stress or cell death to alert and activate the immune system. These DAMPs promote dendritic cell maturation and antigen presentation, leading to an adaptive anti-tumor response. In contrast, the Vjet device suppressed tumors via ROS/RNS-mediated cytotoxicity without initiating an immune response. These findings highlight the utility of the TUM-CAM model in distinguishing between immunologically active and passive anti-cancer mechanisms [90].

Researchers have also transplanted human CRC cell lines such as HT29 and HCT116 into the CAM and investigated the effects of photodynamic therapy on angiogenic factors within the biological microenvironment surrounding CRC cells [96]. Similar experiments have confirmed that drugs targeting STAT1 and PI3K can inhibit CRC angiogenesis, providing insights for new drug development [97,98]. Studies on drug-resistant CRC-CAM models indicate that MEK inhibition may increase radiosensitivity, potentially aiding treatment [99]. In addition to transplanting cancer cell lines, patient-derived tumor tissues can also be grafted. Desette et al. transplanted cancer stem cells from CRC brain metastases (BMSCs) into the CAM, revealing that BMSCs enhanced tumor growth, invasion, and angiogenesis and highlighted the role of BMSCs in metastasis and their potential as therapeutic targets [100].

## 4. Less Commonly Used Mammalian Models for CRC

The earliest documented use of companion animals for oncology dates back to 1915, when Yamagiwa applied coal tar to rabbit ears and observed the onset of skin cancer [101]. Compared with mice and model organisms, livestock mammals (rabbits, dogs, and pigs) exhibit a greater degree of genetic and anatomical similarity to humans. Despite their high costs and stringent ethical considerations, the irreplaceable advantages offered by these models render them indispensable in CRC research.

### 4.1. Pet Rabbits (Oryctolagus cuniculus)

Compared with rodents, pet rabbits are larger animals with a longer lifespan, but are more cost-effective and have a faster reproduction rate. The lifetime cancer risk ranges from 0.5% to 2.7% [102], with an incidence of intestinal adenoma of less than 0.1% [103]. Aside from Yamagiwa’s exploration in 1915, we found no subsequent literature reporting the use of chemical induction or gene editing to construct rabbit cancer models.

As a consequence, most rabbit models are transplantation models, with VX2 tumor transplantation being representative [104]. The VX2 cell line is a squamous cell carcinoma cell line derived from a rabbit papilloma induced by the Shope virus [105]. Depending on experimental needs, these cells can be implanted in different locations to construct various cancer models, such as breast cancer models [106] and liver cancer models [107]. The cells can be injected into the submucosa of the rabbit rectum, where they can colonize the intestines within two weeks to form a tumor prone to metastasize to lymphatic tissues [108]. The rabbit VX2 CRC model is used in preclinical research. For example, a newly developed quantitative high-frequency endoscopic ultrasound has shown great potential in detecting early CRC in VX2 CRC rabbits [108]. Sun et al. compared CT images and gross samples from VX2 CRC rabbits and established a relevant model to assist in the CT diagnosis of early lymph node metastasis in patients with CRC [109]. Gao et al. used these rabbits to investigate the optimal dosage of hematoporphyrin monomethyl ether (HMME) in endoscopic photodynamic therapy (EPDT) for the treatment of CRC and evaluated its efficacy and adverse effects [110]. Researchers are now attempting to genetically modify VX2 cell lines to achieve various experimental objectives. For example, green fluorescent protein genes have been transfected into VX2 cells to better identify cancer cells [111]. However, since VX2 cells are not CRC cell lines, this may affect the relevance of the research outcomes. Researchers have attempted to inject the human CRC cell line HT29 into rabbit livers, and after 8 weeks, they successfully observed tumor growth, thus establishing a humanized rabbit model of liver-metastasized CRC. Nevertheless, this model results in slower growth (for the same total number of injected cells, the diameter of the human-derived CRC was only 0.6 cm after eight weeks, whereas it was greater than 1 cm for the VX2 model within two weeks), which is likely related to immune rejection [112]. We regard this humanized metastasis model as having great potential for the study of CRC liver metastasis and the development of new diagnostic and therapeutic methods.

### 4.2. Pet Dog (Canine)

Dogs are the most common household pets, with a cancer incidence rate five times higher than that of humans [113]. In 2003, the U.S. National Cancer Institute launched the Comparative Oncology Program (COP), which attracted the attention of many countries [114].

The most prevalent model for studying dog CRC is the spontaneous model [115]. A long-term cohort study revealed that the colon and rectum are the primary sites of digestive system tumors in dogs. These tumors exhibit a similar disease process, clinical presentation, morphological features, and prognosis to those in humans [116]. By applying experimental techniques such as liquid biopsy, single-cell transcriptomics, and spatial transcriptomics, along with analytical methods such as nCounter and dependency mapping to canine cancers, researchers have identified numerous new cancer biomarkers, novel mutations, and potential therapeutic targets [113,114,117,118].

Through RNA-Seq technology, researchers have identified three modes of invasion in dog CRC. Two of these models have previously been identified in human CRC, while the third, a crypt-like invasion process, was subsequently shown to also occur in human CRC [115]. Dog CRC also shares a similar tumor immune microenvironment with humans. For example, pathological analysis of dog CRC revealed a consistent alteration in the proportion of immune cells in the tumor tissue, with a reduced proportion of CD18+ and CD3+ cells exerting antitumor effects, mirroring findings in human cases [119]. Furthermore, the gut microbiomes of dogs and humans show a high degree of similarity, and similar bacterial phyla (such as Bacteroidetes, Proteobacteria, and Firmicutes) are enriched within CRC tumors of both species [115,120]. However, it is also important to acknowledge that certain aspects of CRC differ between dogs and humans. For example, while Wnt/β-catenin dysregulation is common in human and dog CRC, the drivers differ: >90% of human CRCs have *APC* mutations, whereas >60% of canine tumors harbor CTNNB1 mutations that mimic *APC* loss by stabilizing β-catenin. Notably, *APC* mutations, hallmarks of human CRC, are rare in dogs [115,121]. Therefore, when utilizing dog CRC models, it is essential to adhere to the principles of comparative biology, considering both similarities and differences.

### 4.3. Domestic Pigs (Sus scrofa domesticus)

Pigs are among the mammals most similar to humans in terms of genetic aspects and disease development. Their genome size is only 7% smaller than that of humans (whereas genomes from mice and dogs are approximately 14% smaller), and they share extensive homology with the human genome [122]. At the nucleotide level, the similarity between pigs and humans is three times greater than that between mice and humans [123]. Additionally, unlike rodents, most pig breeds are outbred and more closely resemble the human population [124]. Pigs have many diseases similar to those of humans, such as hypercholesterolemia [125] and neurological disorders [126]. The application of pigs in oncology research started late but has progressed rapidly. The main modeling methods include induction, gene engineering, and transplantation [127]. Various pig cancer models have been developed, including the N-nitroso-dimethylamine-induced liver cancer model [128], the *TP53* knockout osteosarcoma model [129], and the U87 GM cell line transplant glioblastoma model [130].

Pigs have a digestive tract structure similar to that of humans, making them excellent models for treating CRC. The most classic model is the FAP model, which mimics the *APC* mutation found in humans [131]. Early signs of CRC development, such as loss of cell polarity in polyp tissues, nucleolar enlargement, and activation of the Wnt signaling pathway [131], can be observed in these pigs. Another is the Oncopig Cancer Model, which involves Cre recombinase-induced mutations of *KRASG12D* and *TP53R167H* [132]. Recent studies have reported that CRISPR-Cas9 technology can edit multiple genes simultaneously, inducing lung cancer in pigs, which suggests the potential for constructing pigs with multiple gene mutations in CRC [133]. To better simulate the human CRC environment, researchers have used severe combined immunodeficient (SCID) pigs [134] and engrafted human CD34+ cells or fetal bone marrow thymus to create humanized immune pig models [135]. These CRC models can also be utilized in research on diagnostic techniques and the development of new therapies. Microwave endoscopy, in particular, has shown great potential in the FAP pig model, indicating its possible future application in humans [136].

## 5. Advanced Engineering and Computational Approaches in CRC Modeling

Despite the non-rodent animal models already emerging (Table 1), the deepening of CRC research is driving the need for ideal future tumor models that closely mimic human biology, minimize live animal use, and reduce time and cost. In response to these demands, tumor organoid technology and digital tumor modeling are rapidly developing with the support of novel technologies such as 3D bioprinting, bioengineering, and deep learning.

Organoid technology allows cancer tissue to be obtained from patients and cultured in vitro in 3D to mimic CRC in vivo. Recent technology has even allowed the mimicking of cancer genesis in organoids. Woods et al., through the application of 4-hydroxytamoxifen (4-OHT) and CRISPR/Cas9 gene editing in wild-type organoids, successfully induced CRC development [139]. Researchers have also constructed a healthy, lumen-like structure with a crypt model, the ‘mini-colon’ model, which does not need to be passed on and is capable of inducing spontaneous CRC at a predetermined site. Researchers have confirmed the effect of glutathione peroxidase 2 (GPX2) on CRC formation in “mini colons” [140]. However, these models still have limitations: the tumor microenvironment is progressively disrupted with passaging [141,142]. Moreover, although organoids demonstrate promising accuracy (sometimes over 80%), their predictive power for drug efficacy remains inferior to that of in vivo models because of the absence of critical physiological components [143,144].

Moreover, digital models of tumorigenesis, which are constructed on vast datasets obtained from experiments, are being analyzed and modeled via deep learning algorithms, are emerging [145]. Such models can simulate changes in the molecular characteristics of individual cells and the tumor microenvironment during tumor growth [146], predict potential drug targets [147], and assess the efficacy and side effects of antitumor drugs [148]. Some researchers have high expectations for these models because they are built on extensive data, operate quickly, are cost-effective, and require only computational resources, potentially replicating nearly all the functions of animal models.

## 6. Discussion

As a popular model for cancer research, rodent CRC models’ accessibility, operability, and moderate cost make them widely used. However, their limitations are equally significant. In large-scale experimental studies, rodent models face constraints such as ethical considerations and breeding period, and costs. In translational research, rodent models sometimes fail to perform ideally due to interspecies immunological and metabolic differences. For instance, the combination of immune checkpoint inhibitors, anti-CTLA-4 and anti-PD-1, which demonstrated significant efficacy in mouse models, failed to achieve expected outcomes in clinical trials [149,150]. However, no-rodent species diversity offers distinct advantages and limitations compared to rodents (Table 2), providing researchers with expanded options. Through the application of these models, scientists have achieved numerous translational breakthroughs in elucidating the CRC pathogenic mechanisms, diagnostic techniques, and therapeutic strategies.

Nonrodent models exhibit etiological and mechanistic similarities to human CRC [67]. They have helped researchers discover carcinogens, such as aflatoxins and polycyclic aromatic hydrocarbons (PAHs), and explore their dose–toxicity relationships and their carcinogenic mechanisms [137,138,151]. With progress in molecular biology, researchers have resolved CRC-related signaling pathways and regulatory factors using non-rodent models with smaller, conserved genomes, such as the Hippo/YAP and Hedgehog pathways in the fruit fly and the intestinal epithelial regulatory molecule retinoic acid in zebrafish [30,152,153]. Advances in technologies like gene editing have further expanded the potential of non-rodent models in fundamental and translational research. A prime example comes from fruit fly research, which confirmed that in an *APC*-deficient context, Ras signaling is essential for both tumor initiation and progression, operating in a parallel relationship with the Wnt pathway [54]. These finding challenges traditional linear models, revealing the necessity of targeting Wnt and Ras signaling simultaneously in CRC, providing critical theoretical insights for developing combination therapies. Furthermore, non-rodent models established via CRISPR–Cas9 gene editing can mimic human-specific genetic susceptibilities and anatomical features, facilitating CRC research [136]. For instance, an edited APC mutant pig model of familial adenomatous polyposis (FAP) closely mimics human CRC in anatomy and tumor progression, facilitating evaluation of novel endoscopic diagnostics and minimally invasive surgical techniques [154]. Non-rodent animals also hold irreplaceable value in CRC metastasis and drug resistance research: analysis of dog CRC identified three distinct tumor infiltration patterns, enabling the reclassification of human CRC genomic data into corresponding subtypes and revealing previously unrecognized disease heterogeneity [115], rabbit liver metastasis models help elucidate the formation of the liver niche microenvironment in CRC [112]; while *UPP1* mutant *C. elegans* have become a crucial platform for studying 5-fluorouracil resistance mechanisms [84].

These findings demonstrate that non-rodent models constitute an indispensable component of CRC research. The researcher’s deepening understanding of both rodent and non-rodent models will advance the establishment of a comprehensive research framework encompassing carcinogenic risk assessment, diagnostic validation, elucidation of resistance mechanisms, and innovative therapeutic development (Table 2).

The table systematically contrasts traditional rodent models (mouse, rat, and hamster), large non-rodent species (pig, dog, and non-human primate), and small model organisms (e.g., zebrafish), alongside emerging organoid and digital (computational) systems. Parameters compared include genomic similarity to humans, intestinal anatomy and physiology, experimental cost and manipulability, relevance to metastasis, immune system characteristics, ethical considerations, and appropriate research applications. Rodent models remain fundamental for mechanistic CRC studies due to their genetic tractability and reproducibility, whereas large non-rodent models provide closer physiological and immunological resemblance to humans, enhancing translational relevance. Small model organisms such as zebrafish enable real-time visualization of tumor growth and metastasis at low cost, supporting high-throughput genetic and pharmacological screening. Organoid models derived from patient tissues accurately reproduce epithelial and genetic features of CRC but lack vascular and immune components, while digital models enable virtual simulation of tumor progression, immune interactions, and drug response. Collectively, these complementary in vivo, in vitro, and in silico systems offer a multi-layered framework for mechanistic, translational, and predictive CRC research.

## 7. Future Directions

However, certain systemic limitations inherent in rodent or non-rodent models cannot be fully overcome by simply switching species.

The next breakthrough in CRC model research will not rely on discovering a single perfect model, but on building a new research paradigm, one that designs and applies standardized workflows integrating multiple models according to specific questions. The central challenge, therefore, shifts from selecting a single model to creating integrated and scalable research pathways. The foundation of this lies in promoting deep association among rodent, non-rodent, bioengineered, and digital models, forming a loop that connects mechanistic exploration, diagnostic development, and therapeutic innovation. The value of this review is to provide a conceptual framework for this idea, showing how CRC models beyond rodents can complement traditional systems.

First, a functionally complementary pipeline should be established. An effective re-search pathway could begin with high-throughput screening, making use of the efficiency and scalability of non-rodent models (such as zebrafish and fruit fly) and bioengineering platforms (such as organoids) for large-scale drug screening and gene interaction studies. The most promising candidates from this stage can then be tested in rodent models with complete physiological systems (e.g., Genetically Engineered Mouse, GEM) to verify target specificity and in vivo efficacy. Finally, approaches showing strong translational potential should be evaluated in large non-rodent models (such as dogs or pigs), which more closely resemble human physiology and immune contexts. This “screening–validation–confirmation” process helps ensure that each clinical hypothesis is tested in the most relevant and predictive experimental systems. Second, advancing data and platform standardization is crucial to establishing a common language for comparing results across models. Effective collaboration between different model systems requires breaking down data silos and defining unified standards across species and experimental platforms. For instance, standardized experimental and analytical workflows could be developed to generate PDX, organoid, and zebrafish xenograft models in parallel from the same patient sample, followed by evaluation using consistent biomarkers and efficacy measures. Artificial intelligence and computational modeling play a key role here; by integrating standardized datasets, they can help “calibrate” the predictive power of each model and identify disease mechanisms that extend beyond the scope of any single system. Finally, the field should move toward building a highly integrated “digital–physical” predictive research loop. Within such a framework, AI-driven digital twin models can reconstruct tumor behavior based on multi-omics data, enabling virtual testing of therapeutic strategies and reducing unnecessary experimental work. The hypotheses generated computationally can then be verified in organoid and animal models, with the experimental feedback used to refine the digital systems. This iterative cycle, “computational prediction → experimental validation → feedback optimization”, has the potential to greatly improve both the efficiency and success rate of translating basic discoveries into clinical practice.

Eventually, the future of CRC research does not lie in seeking the “ultimate answer” from any single model, but in logically integrating the entire modeling ecosystem. By integrating the mechanistic precision of rodent models, the translational relevance of non-rodent systems, and the predictive capabilities of bioengineered and digital platforms, CRC research can evolve into a more reliable, efficient, and patient-focused pathway for developing precision therapies.

## Figures and Tables

**Table 1:** Colorectal Cancer Non-Rodent Animal Models.

Species	Spontaneous Model	Induced Models	Genetically Engineered Models	Transplant Models
Zebrafish	√ [32]	TNBS Induced Model [33]	APC Mutant Model [40]	hCRC Cell Line Model [31]
(*Danio rerio*)		DSS Induced Model [6]	K-RAS Transgenic Model [41]	PDX Model [31]
		DMBA Induced Model [34]	TP53 Mutant Model [42]	
			RAG2 Mutant Model [44]	
Black-bellied fruit fly	×	×	APC Mutant Model [60]	×
(*Drosophila melanogaster*)			Notch Deficiency Model [61]	
			HPZ multi-gene mutant Model [56]	
*C. elegans*	×	×	UPP-1 mutant Model [88]	×
(*Caenorhabditis elegans*)				
Chorioallantoic Membrane	×	×	×	mouse Cell Line Model [98]
(CAM) Model				hCRC Cell Line Model [102]
				PDX Model [106]
Pet Rabbits	√ [109]	×	×	VX2 Model [113]
(*Oryctolagus cuniculus*)				hCRC Cell Line Model [116]
Pet Dog	√ [125]	×	×	×
*(Canine)*				
Domestic Pigs	×	×	APC Mutant Model [137]	×
(*Sus scrofa domesticus*)			Oncopig Model [138]	

Models can be categorized into spontaneous models, induced models, genetically engineered models (GEMs), and transplantation models. In model organisms, the methods for constructing CRC models are more diverse, but spontaneous CRC is relatively rare. Considering the ethical and cost limitations in mammals, there are fewer artificial tumor models but more spontaneous intestinal cancers. Table 1 √ indicates that the model exists, and × indicates that the model does not exist. The reference is indicated in brackets.

**Table 2:** Comparative overview of models used in CRC research.

Category	Parameter	Mouse	Rat	Hamster	Zebrafish	Dog	Pig	Non-Human Primate	Organoid Models	Digital Models
Genomic and Physiological	Genomic similarity to humans	~85%	~85%	~84%	~70%	~94%	~95%	~98%	≈100% (human-derived)	Model-dependent (data-driven)
	Intestinal anatomy and physiology	Moderate, villus–crypt organization similar but shorter colon	Moderate, longer colon but physiological differences	Limited, thinner mucosa	Low, simple tubular gut, lacks colon	Similar, comparable epithelial cell types	Very similar, comparable colonic length, mucosal structure, mucus secretion	Very similar, conserved crypt–villus structure and mucus profile	Faithfully mimics epithelium; lacks vasculature/immune system	Can replicate systemic physiology virtually
Experimental Practicality	Cost of maintenance	Moderate	Moderate	Moderate	Low	High	High	Very high	Moderate/High	High initial investment, Low marginal cost
	Ease of genetic manipulation	High (e.g., ApcMin, Cdx2, P53 models)	Moderate (e.g., ENU and carcinogen models available)	Limited	High (e.g., CRISPR, transgenic lines)	Limited	Emerging (e.g., CRISPR, APC-mutant pigs)	Limited due to ethics	High (CRISPR editing, co-culture feasible)	Flexible (parameters easily modified)
	Availability of CRC models	Extensive (spontaneous, carcinogen-induced, PDX, GEMMs)	Well-established chemically induced models	Rare; limited spontaneous CRC	Chemical or transgenic tumor models in progress	Limited to spontaneous or induced adenomas	Expanding (APCΔ/Δ pigs, sporadic CRC models)	Few, but closest to human sporadic CRC	Partially recapitulates invasion; lacks circulation	Dependent on modeling accuracy
Relevance to CRC Metastasis	Ability to mimic metastatic cascade	Well-established (liver, lung, lymphatic spread)	Established (liver, peritoneal)	Rare	Limited	Moderate	High translational potential (liver metastasis similar to humans)	Highly representative of human metastatic CRC	Contains only epithelial layer	Integrates virtual immune networks
	Tumor microenvironment resemblance	Moderate, lacks human-like stromal composition	Moderate	Low	Low, lacks adaptive immunity	Moderate	High, immune and stromal cells similar to humans	Very high, highly similar immune–stromal interaction	Absent unless co-cultured	Model-dependent (data-driven)
Immune System	Similarity to human immune response	Moderate	Moderate	Moderate	Low	High	High	Very high	Possible via immune co-culture	Depends on model design
	Humanized models available	Yes	Partial	No	No	No	Emerging (immunocompetent models)	Limited		
Ethical/Logistical	Ethical constraints	Low	Low	Low	Low	High	High	Very high	Very low (no animal use)	None
	Housing and handling	Easy, small space	Easy	Easy	Simple	Complex	Complex; requires specialized facilities	Complex and expensive	Moderate (standard culture facilities)	Simple (platform-based)
Suitable Research Applications	—	Mechanistic CRC studies, gene–environment interactions, immunotherapy	Carcinogen-induced CRC, chemoprevention, pharmacokinetics	Early tumorigenesis, infection-related CRC	High-throughput screening, angiogenesis	Toxicity and pharmacology	Translational CRC pathology, surgery, preclinical drug testing	Translational validation, immune and metastatic modeling	Precision oncology, drug response, patient stratification	Predictive modeling, drug discovery, clinical outcome simulation
Limitations vs. Human CRC	—	Small size, faster tumor progression, simplified microbiota	Limited genetic tools	Poor physiological similarity	Simplified gut structure, immunodeficiency	Cost, limited accessibility	High cost, ethical limits, limited resources	Ethical barriers, limited sample size	Lacks vascular, immune, and microbiota components	Abstracted biology, dependent on data quality

## Data Availability

No new data were created or analyzed in this study.

## Abbreviations

The following abbreviations are used in this manuscript:

APC	adenomatous polyposis coli
BMP	bone morphogenetic protein
BMSC	brain metastasis stem cells
*C. elegans*	*Caenorhabditis elegans*
CAM	chorioallantoic membrane
COP	Comparative Oncology Program
CRC	colorectal cancer
DMBA	7,12-dimethylbenzanthracene
DSS	dextran sodium sulfate
EGFR	epidermal growth factor receptor
EMT	epithelial–mesenchymal transition
ENU	N-ethyl-N-nitrosourea
EOCRC	early-onset colorectal cancer
FAP	familial adenomatous polyposis
FGF	fibroblast growth factor
ISC	intestinal stem cell
MMP	matrix metalloproteinases
PDX	patient-derived xenograft
RAG2	recombination-activating gene 2
SCID	severe combined immunodeficient
TNBS	2,4,6-trinitrobenzene sulfonic acid
TP53	tumor protein p53
TUM-CAM	tumor chorioallantoic membrane
UPP1	uridine phosphorylase 1
5-FU	5-fluorouracil
